# Factors associated with optic disc parameters and circumpapillary retinal nerve fiber layer thickness in 8-year-old children: The Yamanashi Adjunct Study of the Japan Environment and Children’s Study

**DOI:** 10.1371/journal.pone.0330335

**Published:** 2025-08-20

**Authors:** Ryo Harada, Mingxue Bao, Natsuki Okabe, Yuka Kasai, Airi Takahashi, Chio Kuleshov, Yumi Shigemoto, Tadao Ooka, Hiroshi Yokomichi, Kunio Miyake, Reiji Kojima, Ryoji Shinohara, Hideki Yui, Sanae Otawa, Megumi Kushima, Zentaro Yamagata, Kenji Kashiwagi, the Yamanashi Adjunct Study of the Japan Environment and Children’s Study Group

**Affiliations:** 1 Department of Ophthalmology, Interdisciplinary Graduate School of Medicine, University of Yamanashi, Yamanashi, Japan; 2 Department of Ophthalmology, Kofu Kyoritsu Clinic, Yamanashi, Japan; 3 Department of Health Sciences, Interdisciplinary Graduate School of Medicine, University of Yamanashi, Yamanashi, Japan; 4 Department of Epidemiology and Environmental Medicine, Interdisciplinary Graduate School of Medicine and Engineering, University of Yamanashi, Yamanashi, Japan; 5 Center for Birth Cohort Studies, Interdisciplinary Graduate School of Medicine, University of Yamanashi, Yamanashi, Japan; Akita University: Akita Daigaku, JAPAN

## Abstract

**Purpose:**

The purpose of this study was to investigate the relationships between the circumpapillary retinal nerve fiber layer thickness (cpRNFL) and optic disc parameters and associated factors in 8-year-old Japanese children who participated in the Yamanashi Adjunct Study of the Japan Environment and Children’s Study (JECS).

**Subjects and methods:**

The participants in this study were 559 8-year-old Japanese children (277 boys and 282 girls) who participated in the Yamanashi Adjunct Study of JECS at the University of Yamanashi from June 2021 to March 2022. The visual acuity, spherical equivalent (SE), and axial length (AL) of the participants were measured. The cpRNFL thickness was measured with a spectral domain optical coherence tomograph (NIDEK RS-3000 Advance, Gamagori, Japan).

**Results:**

The data of 349 participants (182 boys and 167 girls) were ultimately analyzed. Multivariable analysis showed that AL was significantly positively correlated with the cpRNFL thickness in the 8 o’clock sector (β = 0.26; 95% confidence interval (CI) 2.74–6.87) and significantly negatively correlated with the cpRNFL thickness in the 6 o’clock sector (β = −0.21; 95%CI −9.94–-2.98). The cpRNFL thickness in the 10 o’clock and 11 o’clock sectors was greater for girls than for boys (β = 0.21; 95%CI 2.54–8.34; and β = 0.15; 95%CI 1.85–11.99, respectively), whereas in the 12 o’clock sector, it was greater for boys (β = −0.26; 95%CI −20.41–-8.32). The disc area was significantly positively correlated with the cpRNFL thickness in the 7 o’clock, 10 o’clock and 11 o’clock sectors (β = 0.13, 95%CI 0.93–8.67; β = 0.13, 95%CI 10.36–23.88; and β = 0.27, 95%CI 10.36–23.88, respectively).

**Conclusion:**

The thickness of the cpRNFL in 8-year-old Japanese children was associated with AL, sex, and disc area, but these associations differed by RNFL sector.

## Introduction

Optical coherence tomography (OCT) is often used in pediatric ophthalmology because it can noninvasively evaluate the retina and circumpapillary retinal nerve fiber layer (cpRNFL) and rapidly acquire high-resolution images, even for children who cannot cooperate well with the examination [1,2].

According to the many reports on the factors affecting the cpRNFL thickness, it is generally agreed that in adults, the cpRNFL thickness decreases with increasing axial length (AL) and degree of myopia [3]. In a report by Zha et al. involving subjects aged 7–35 years, compared with that of subjects in the low-moderate myopia group, the retinal nerve fiber layer of subjects in the high myopia group (−6 D or higher) was thicker in the temporal quadrant and thinner in the superior, nasal, and inferior quadrants [4]. In a study involving children, Lim reported a significantly thinner cpRNFL thickness in the strongly (−6 D or higher) myopic group than in the mildly (−3 D or less) myopic group [5]. Racial differences in the cpRNFL thickness have also been reported, with one report showing a decrease by 2.2 µm when the AL increased by 1 mm in Caucasian children but no changes in African American children [6]. In terms of potential sex differences, Zhang et al. reported that the whole retinal nerve fiber layer was thicker in girls than in boys [7], but some reports have also identified no sex differences in children [8,9]. Although it is important to consider factors affecting the cpRNFL thickness when investigating the pathogenesis of optic nerve diseases such as glaucoma, there have been fewer reports of large-scale studies in children than in adults, and no consensus has been reached regarding the effects of AL, refractive error, age, and sex on the thickness of the cpRNFL [3]. In addition, few large-scale studies specifically focused on Japanese children have been conducted.

The purpose of this study was to examine the relationships between cpRNFL thickness and AL, refractive error, sex, and various optic disc parameters in 8-year-old children.

## Materials and methods

### Study design

This study was conducted as part of the Yamanashi Adjunct Study of the Japan Environment and Children’s Study (JECS) [10,11]. The JECS is an ongoing nationwide birth cohort study, a national project that aims to determine the impact of environmental factors on child health from the fetal period to adulthood. The study includes more than 100,000 pregnant women who were enrolled in 15 JECS study areas across Japan between January 2011 and March 2014.The JECS protocol and baseline data have been detailed in a previous report [12,13]. The JECS conducts school-age examinations for all participants, including physical measurements, blood tests, urinalysis, and developmental examinations at the ages of 8 and 12. The Yamanashi Adjunct Study of JECS is a comprehensive health examination conducted independently by the University of Yamanashi for those who wish to participate. This study includes ophthalmologic examinations, dental examinations, physical fitness tests, questionnaire surveys, and other health examination items. The JECS conducts a survey every four years after birth, and the children in this study were 8 years old. The participants in this study were 559 8-year-old Japanese children (277 boys and 282 girls) who participated in the Yamanashi Adjunct Study of JECS at the University of Yamanashi from June 2021 to March 2022. Only data from the Yamanashi Adjunct Study of JECS were used in this study.

### Ethics statement

The JECS protocol was reviewed and approved by the Ministry of the Environment’s Institutional Review Board on Epidemiological Studies. This study was approved by the Ethical Review Committee of the University of Yamanashi School of Medicine. This study was conducted in accordance with the ethical guidelines and regulations of the Declaration of Helsinki. Written informed consent was obtained from all participants and parents of children prior to their participation in the study.

### Examination

Ophthalmologic examinations were performed to measure the uncorrected visual acuity, corrected visual acuity, AL, and objective refraction, as detailed in Okabe et al. [10], as well as the cpRNFL thickness and optic nerve head shape. Visual acuity was measured with a 3-meter visual acuity chart. AL was measured with an optical biometer (NIDEK AL-SCAN, Gamagori, Japan). An autorefractometer (NIDEK AR-F, Gamagori, Japan) was used to measure the objective refractive error without the use of cycloplegic drops, and the spherical equivalent (SE) was calculated from the mean value of three measurements. Spectral domain optical coherence tomography (SD-OCT) (NIDEK RS-3000 Advance2, Gamagori, Japan) was used to measure the cpRNFL thickness and optic disc parameters. The ophthalmologic examinations were performed by ophthalmologists and certified orthoptists.

### OCT measurements

**Fig 1:**
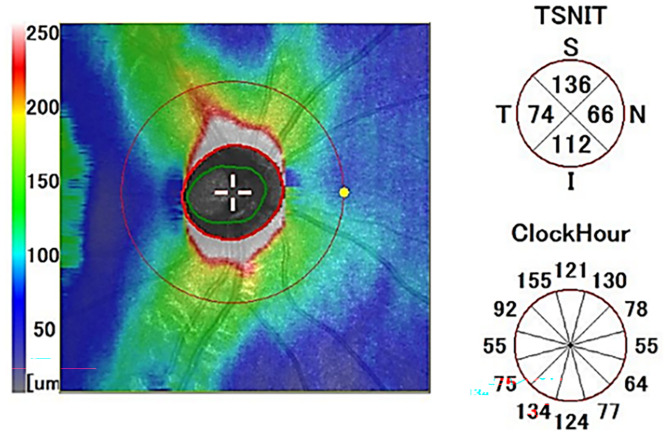
Measurement of circumpapillary retinal nerve fiber layer.

The RS-3000 Advance2 SD-OCT has a light source wavelength of 880 nm and a resolution of 20㎛ in the lateral direction and 7㎛ in the depth direction; it can perform 85,000 A-scans per second. A disc map scan with an area of 6 × 6 mm^2^ centered on the optic nerve head was performed, and a 512 A scan×128 B scan was used to measure the cpRNFL thickness, disc area, cup area, and cup-to-disc ratio (CDR). The semiautomatic segmentation program of the NAVIS-EX image analysis software was used to analyze the thickness of the cpRNFL from the inner limiting membrane to the retinal ganglion cell layer as well as the disc area and cup area. The cpRNFL thickness was assessed in terms of the mean thickness, the thickness in the four quadrants (temporal, superior, nasal, inferior), and the thickness in 12 sectors from 1 o’clock–12 o’clock (Fig 1) [14]. To correct for ocular magnification effects [15], the AL correction routine in NAVIS-EX was employed, and image analysis was performed after the AL values were entered for each participant.

### Inclusion criteria

**Fig 2:**
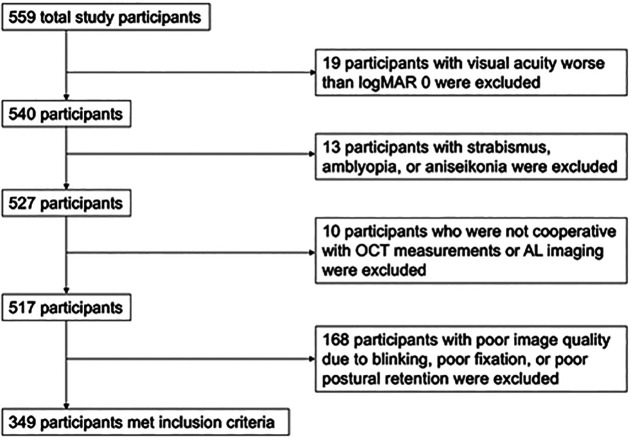
Inclusion and exclusion flow chart. Abbreviations: optical coherence tomography (OCT), axial length (AL).

The participant inclusion criteria were as follows: a corrected visual acuity of logMAR 0 or better and no strabismus, amblyopia, or aniseikonia. The inclusion criteria for the eye imaging characteristics were as follows: a signal-to-noise ratio for the AL measurement greater than 5; an OCT image scan quality index greater than 2; a scan signal index greater than 7; and good fixation during imaging. The right eye was used for the measurements whenever possible; the left eye was used only when the right eye was unavailable. When the left eye was used, it was flipped and analyzed so that the temporal and nasal directions matched those of the right eye. Participants were not asked whether they had eye or systemic diseases. Nineteen participants with a visual acuity worse than logMAR 0 and 13 participants with strabismus, amblyopia, or aniseikonia were excluded. In addition, 10 participants who were not cooperative during the OCT measurements or AL imaging and 168 participants whose images were of poor quality due to blinking, poor fixation, or poor postural retention were excluded. A total of 349 eyes of 349 participants (182 boys and 167 girls) met the inclusion criteria and were included in the final analysis (Fig 2). Of the 349 who met the inclusion criteria, 213 used their right eye and 136 used their left eye.

### Statistical analysis

EZR for Windows (Jichi Medical University, Japan) [16] was used for the statistical analyses. Differences in the OCT measurements among the four sectors (temporal, superior, nasal, inferior) and differences in AL, SE, and logMAR between the included and excluded groups were analyzed with one-way ANOVA with Bonferroni correction. Single regression analyses between the AL, SE and cpRNFL thickness and the optic disc parameters were performed with Pearson’s product‒moment correlation coefficient. Sex differences in visual acuity, AL, SE, cpRNFL thickness, and optic disc parameters were analyzed using Student’s t test for independent groups. Multivariable regression analysis was performed using cpRNFL thickness, optic disc parameters, AL, and sex as variables of interest, with Bonferroni correction applied. The difference in cpRNFL thickness between the left and right eyes of the included group was analyzed with the Mann-Whitney U test. The sex ratios of the included and excluded groups were compared with the χ^2^ test. For each analysis, a P value of less than 5% was considered to indicate statistical significance.

## Results

### Demographics

Table 1 shows the mean values of the AL, SE, and uncorrected logMAR for the 349 participants in the included group. Boys had a significantly greater AL than girls (P < 0.001).

**Table 1:** AL, SE, and logMAR of participants in the included group.

	All participants (n = 349)	Boys (n = 182)	Girls (n = 167)	P^a^
AL (mm)	23.11 ± 0.77 (21.90–24.37)	23.36 ± 0.72 (22.25–24.46)	22.83 ± 0.73 (21.68–24.05)	<0.001
SE (D)	−0.35 ± 0.94 (−1.88– + 0.63)	−0.44 ± 0.85 (−1.88– + 0.51)	−0.25 ± 1.03 (−1.74– + 0.88)	0.05
Uncorrected logMAR	0.06 ± 0.18 (0.00–0.52)	0.06 ± 0.19 (0.00–0.52)	0.06 ± 0.18 (0.00–0.49)	0.80

The data are presented as the means ± standard deviations (5th‒95th percentiles).

Abbreviations: axial length (AL), spherical equivalent (SE).

^a^Student’s t tests.

### Circumpapillary retinal nerve fiber layer thickness and optic disc measurements

The mean thicknesses of the cpRNFL and the disc area, cup area, and CDR are shown in Table 2. The cpRNFL was thickest in the superior quadrant, followed by the inferior, temporal, and nasal quadrants. There was no significant difference between the thickness in the superior and inferior quadrants, but significant differences were found between all other pairs of quadrants (P < 0.001) (S1 Table).

**Table 2:** Mean thickness of the cpRNFL and the mean values of optic disc parameters.

Parameters	All participants (n = 349)
cpRNFL Thickness (µm)	
Whole	96.1 ± 9.0 (81–111)
Temporal	74.2 ± 9.6 (60–90)
Superior	124.2 ± 16.3 (96–151)
Nasal	62.5 ± 11.1 (46–82)
Inferior	123.5 ± 14.7 (100–147)
1 o’clock	108.5 ± 23.5 (69–145)
2 o’clock	75.5 ± 18.7 (49–107)
3 o’clock	50.3 ± 8.8 (38–67)
4 o’clock	61.6 ± 14.4 (41–88)
5 o’clock	94.1 ± 19.6 (64–128)
6 o’clock	130.6 ± 23.4 (92–168)
7 o’clock	145.6 ± 22.5 (107–182)
8 o’clock	75.3 ± 14.3 (55–103)
9 o’clock	60.1 ± 9.8 (48–76)
10 o’clock	87.0 ± 13.3 (66–108)
11 o’clock	141.8 ± 23.4 (101–177)
12 o’clock	122.6 ± 27.8 (80–172)
Optic disc parameters	
Disc area (mm^2^)	2.07 ± 0.37 (1.52–2.71)
Cup area (mm^2^)	0.56 ± 0.31 (0.15–1.11)
CDR	0.26 ± 0.12 (0.09–0.47)

The data are presented as the means ± standard deviations (5th‒95th percentiles).

Abbreviations: circumpapillary retinal nerve fiber layer thickness (cpRNFL), cup-to-disc ratio (CDR).

### Single regression analysis

A single regression analysis of cpRNFL, disc parameters, and AL is shown in Table 3. AL was significantly positively correlated with cpRNFL thickness at 8 o’clock (R = 0.28, 95%CI 0.18–0.37) and 9 o’clock (R = 0.11, 95%CI 0.001–0.20) sectors, and significantly negatively correlated with cpRNFL thickness at 6 o’clock sector (R = −0.15, 95%CI −0.24–-0.04). The disc area (R = 0.29, 95%CI 0.19–0.39) and cup area (R = 0.17, 95%CI 0.07–0.27) were significantly positively correlated with AL.

**Table 3:** Single regression analysis of cpRNFL, disc parameters, and AL.

	AL			
	R	95%CI		P
cpRNFL thickness				
Whole	0.06	−0.04	0.17	0.25
1 o’clock	0.10	−0.009	0.19	0.07
2 o’clock	0.0005	−0.10	0.10	0.99
3 o’clock	−0.02	−0.12	0.08	0.72
4 o’clock	0.03	−0.07	0.13	0.54
5 o’clock	−0.07	−0.17	0.03	0.21
6 o’clock	−0.15	−0.24	−0.04	0.006
7 o’clock	−0.02	−0.12	0.08	0.73
8 o’clock	0.28	0.18	0.37	<0.001
9 o’clock	0.11	0.001	0.20	0.04
10 o’clock	0.09	−0.01	0.19	0.09
11 o’clock	0.009	−0.09	0.11	0.86
12 o’clock	0.10	−0.008	0.20	0.07
Optic disc parameters				
Disc area	0.29	0.19	0.39	<0.001
Cup area	0.17	0.07	0.27	0.001
CDR	0.10	−0.01	0.20	0.06

Abbreviations: circumpapillary retinal nerve fiber layer thickness (cpRNFL), axial length (AL), correlation coefficient (R), confidence interval (CI), cup-to-disc ratio (CDR).

A single regression analysis of cpRNFL, disc parameters, and SE is shown in Table 4. SE was significantly negatively correlated with cpRNFL thickness at 8 o’clock sector (R = −0.16, 95%CI −0.26–-0.05) and significantly positively correlated with cpRNFL thickness at 6 o’clock sector (R = 0.15, 95%CI 0.04–0.25). The disc area was significantly negatively correlated with SE (R = −0.16, 95%CI −0.26–-0.05).

**Table 4:** Single regression analysis of cpRNFL, disc parameters, and SE.

	SE			
	R	95%CI		P
cpRNFL thickness				
Whole	−0.01	−0.11	0.09	0.90
1 o’clock	−0.07	−0.17	0.03	0.17
2 o’clock	0.04	−0.06	0.15	0.41
3 o’clock	0.06	−0.04	0.16	0.27
4 o’clock	0.08	−0.02	0.18	0.12
5 o’clock	0.09	−0.01	0.19	0.09
6 o’clock	0.15	0.04	0.25	0.004
7 o’clock	−0.04	−0.14	0.06	0.45
8 o’clock	−0.16	−0.26	−0.05	0.002
9 o’clock	−0.05	−0.15	0.05	0.33
10 o’clock	−0.02	−0.12	0.08	0.75
11 o’clock	−0.10	−0.20	0.008	0.07
12 o’clock	−0.02	−0.12	0.08	0.68
Optic disc parameters				
Disc area	−0.16	−0.26	−0.05	0.003
Cup area	−0.05	−0.16	0.06	0.34
CDR	−0.009	−0.11	0.10	0.87

Abbreviations: circumpapillary retinal nerve fiber layer thickness (cpRNFL), spherical equivalent (SE), correlation coefficient (R), confidence interval (CI), cup-to-disc ratio (CDR).

### Sex differences in the OCT measurements

The sex differences in the cpRNFL thickness and optic disc parameters are summarized in Table 5. Boys had a thicker cpRNFL in the 1 o’clock (P = 0.006) and 12 o’clock (P < 0.001) sectors, and girls had a thicker cpRNFLs in the 10 o’clock (P = 0.006) and 11 o’clock (P = 0.02) sectors. The disc area was significantly larger for boys than for girls (P = 0.01).

**Table 5:** Optical coherence tomography measurements by sex.

	Boys (n = 182)	Girls (n = 167)	P^a^
cpRNFL thickness (µm)			
Whole	96.7 ± 9.4 (79–112)	95.4 ± 8.4 (82–109)	0.16
1 o’clock	111.8 ± 23.6 (71–147)	105.0 ± 23.0 (67–144)	0.006
2 o’clock	76.4 ± 19.6 (49–116)	74.6 ± 18.0 (49–103)	0.36
3 o’clock	50.3 ± 9.0 (38–67)	50.3 ± 8.6 (38–66)	0.98
4 o’clock	61.9 ± 15.1 (41–91)	61.3 ± 13.7 (43–86)	0.71
5 o’clock	94.4 ± 20.8 (63–131)	93.9 ± 18.3 (67–127)	0.80
6 o’clock	131.3 ± 25.2 (90–173)	129.8 ± 21.4 (95–165)	0.55
7 o’clock	143.8 ± 21.0 (108–176)	147.5 ± 24.0 (106–190)	0.12
8 o’clock	76.3 ± 14.8 (55–104)	74.2 ± 13.5 (56–101)	0.15
9 o’clock	60.6 ± 9.4 (48–79)	59.6 ± 10.2 (48–74)	0.33
10 o’clock	85.1 ± 13.4 (66–106)	89.0 ± 12.9 (68–114)	0.006
11 o’clock	139.2 ± 22.7 (101–173)	144.6 ± 23.9 (102–178)	0.02
12 o’clock	129.6 ± 28.3 (86–178)	114.9 ± 25.2 (79–156)	<0.001
Optic disc parameters			
Disc area (mm^2^)	2.12 ± 0.37 (1.54–2.82)	2.02 ± 0.36 (1.50–2.62)	0.01
Cup area (mm^2^)	0.58 ± 0.33 (0.15–1.20)	0.53 ± 0.29 (0.15–1.07)	0.09
CDR	0.27 ± 0.12 (0.09–0.47)	0.26 ± 0.12 (0.09–0.46)	0.40

The data are presented as the means ± standard deviations (5th‒95th percentiles).

Abbreviations: circumpapillary retinal nerve fiber layer thickness (cpRNFL), cup-to-disc ratio (CDR).

^a^Student’s t tests.

### Multivariable regression analysis

The results of the multivariable regression analysis with the cpRNFL thickness as the dependent variable and AL, sex, and the disc area as the independent variables are summarized in Table 6. In the multivariable regression analysis, sex was converted into a numerical variable by assigning boys a value of 0 and girls a value of 1.

**Table 6:** Relationship between cpRNFL thickness, AL, sex, and disc area.

cpRNFL Thickness	AL					Sex					DIsc area				
	B^a^	95%CI of B		β^b^	P	B^a^	95%CI of B		β^b^	P	B^a^	95%CI of B		β^b^	P
1 o’clock	0.84	−2.68	4.35	0.03	1.00	−5.78	−11.00	−0.55	−0.12	0.09	5.56	−1.40	12.53	0.09	0.33
2 o’clock	−1.25	−4.07	1.58	−0.05	1.00	−1.88	−6.07	2.32	−0.05	1.00	5.96	0.37	11.56	0.12	0.09
3 o’clock	−0.02	−1.35	1.31	−0.001	1.00	−0.21	−2.18	1.77	−0.01	1.00	−1.74	−4.37	0.90	−0.07	0.57
4 o’clock	0.27	−1.92	2.46	0.01	1.00	−0.21	−3.46	3.04	−0.01	1.00	2.05	−2.28	6.39	0.05	1.00
5 o’clock	−2.32	−5.30	0.65	−0.09	0.36	−1.54	−5.96	2.88	−0.04	1.00	2.08	−3.81	7.98	0.04	1.00
6 o’clock	−6.46	−9.94	−2.98	−0.21	0.003	−4.13	−9.30	1.05	−0.09	0.33	7.84	0.95	14.74	0.12	0.06
7 o’clock	−0.73	−4.11	2.64	−0.03	1.00	4.11	−0.91	9.12	0.09	0.30	7.97	1.28	14.66	0.13	0.03
8 o’clock	4.80	2.74	6.87	0.26	<0.001	0.80	−2.27	3.87	0.03	1.00	4.07	−0.03	8.17	0.11	0.15
9 o’clock	1.11	−0.37	2.59	0.09	0.39	−0.31	−2.51	1.88	−0.02	1.00	1.16	−1.76	4.09	0.04	1.00
10 o’clock	2.08	0.12	4.03	0.12	0.09	5.44	2.54	8.34	0.21	<0.001	4.80	0.93	8.67	0.13	0.03
11 o’clock	−0.56	−3.97	2.85	−0.02	1.00	6.92	1.85	11.99	0.15	0.01	17.12	10.36	23.88	0.27	<0.001
12 o’clock	−0.37	−4.43	3.70	−0.01	1.00	−14.36	−20.41	−8.32	−0.26	<0.001	4.58	−3.48	12.64	0.06	0.78

Sex was analyzed by assigning boys a value of 0 and girls a value of 1.

Abbreviations: circumpapillary retinal nerve fiber layer thickness (cpRNFL), axial length (AL), confidence interval(CI).

^a^Nonstandardized Regression Coefficient B.

^b^Standardized Regression Coefficient β.

The cpRNFL thickness in the 6 o’clock sector (β = −0.21, 95%CI −9.94–-2.98) was significantly negatively correlated with AL, whereas that in the 8 o’clock sector (β = 0.26, 95%CI 2.74–6.87) was significantly negatively correlated with AL.

The thickness of the cpRNFL in the 10 o’clock (β = 0.21, 95%CI 2.54–8.34) and 11 o’clock (β = 0.15, 95%CI 1.85–11.99) sectors was significantly positively correlated with sex, whereas that in the 12 o’clock sector (β = −0.26, 95%CI −20.41–-8.32) was significantly negatively correlated with sex.

The thickness of the cpRNFL in the 7 o’clock (β = 0.13, 95%CI 1.28–14.66), 10 o’clock (β = 0.13, 95%CI 0.93–8.67) and 11 o’clock (β = 0.27, 95%CI 10.36–23.88) sectors were significantly positively correlated with the disc area.

Table 7 summarizes the results of the multivariable regression analysis in which disc area, cup area and CDR were used as the dependent variables and AL, sex, and the thickness of the whole cpRNFL were used as the independent variables. The disc area was significantly positively correlated with the AL (β = 0.27, 95%CI 0.08–0.18) and whole cpRNFL thickness (β = 0.19, 95%CI 0.003–0.01), and the cup area was significantly positively correlated with AL (β = 0.16, 95%CI 0.02–0.11).

**Table 7:** Relationships between optic disc parameters and AL, sex, and whole cpRNFL thickness.

	AL					Sex					Whole cpRNFL thickness				
	B^a^	95%CI of B		β^b^	P	B^a^	95%CI of B		β^b^	P	B^a^	95%CI of B		β^b^	P
Disc area	0.13	0.08	0.18	0.27	<0.001	−0.02	−0.10	0.06	−0.03	1.00	0.01	0.003	0.01	0.19	<0.001
Cup area	0.06	0.02	0.11	0.16	0.01	−0.02	−0.09	0.05	−0.03	1.00	0.0006	−0.003	0.004	0.02	1.00
CDR	0.02	−0.003	0.03	0.09	0.27	−0.004	−0.03	0.02	−0.02	1.00	−0.0005	−0.002	0.001	−0.03	1.00

Sex was analyzed by assigning boys a value of 0 and girls a value of 1.

Abbreviations: axial length (AL), circumpapillary retinal nerve fiber layer thickness (cpRNFL), confidence interval (CI), cup-to-disc ratio (CDR).

^a^Nonstandardized Regression Coefficient B.

^b^Standardized Regression Coefficient β.

## Discussion

This work was a cross-sectional study involving 8-year-old Japanese children. The cpRNFL was thinner in the 6 o’clock sector and thicker in the 8 o’clock sector because of axial elongation and myopia. Additionally, a sex difference in the cpRNFL thickness was identified, with girls having a thicker cpRNFL than boys at 10 o’clock and 11 o’clock and boys having a thicker cpRNFL than girls at 12 o’clock. Moreover, the cpRNFL thickness increased as the disc area increased. Unlike previous reports, in this study, the thickness of the cpRNFL was analyzed in 12 clock position-related sectors, and the optic disc was included in the analysis.

Comparisons of our findings with those of previous reports are summarized in Table 8 and 9.

**Table 8:** Comparison of cpRNFL thickness in children across studies.

	Age	Participants	Race	Equipment	Whole	Temporal	Superior	Nasal	Inferior
	Range	n			Mean±SD (µm) (5th–95th percentile)				
Current Study	8	349	Japanese	RS-3000 Advance2	96.1 ± 9.0 (81–111)	74.2 ± 9.6 (60–90)	124.2 ± 16.3 (96–151)	62.5 ± 11.1 (46–82)	123.5 ± 14.7 (100–147)
Al-Haddad et al [17]	6–18	108	Caucasian Middle Eastern	Cirrus-HD	NA	66 (54–84)	121 (99–145)	70 (49–94)	125 (95–159)
Bario-Bario et al [9]	4–17	281	Caucasian	Cirrus 4000	97.4 ± 9.0 (82.4–113.3)	67.4 (51.8–83.3)	124.7 (98.4–152.0)	69.7 (52.0–89.0)	128 (103.5–154.7)
Bueno-Gimeno et al [18]	6–17	293	Caucasian	Cirrus-HD	99.46 ± 11.21	72.73 ± 16.33	123.63 ± 22.76	70.20 ± 15.07	125.75 ± 23.01
Dave et al [19]	5–18	126	Indian	Spectralis	100.3 ± 8.3	74.1 ± 10.9	125.2 ± 18.3	74.8 ± 13.8	127.2 ± 11.1
Zhang et al [7]	6–8	4034	Chinese	Spectralis	106.60 ± 9.41	NA	NA	NA	NA

Abbreviations: circumpapillary retinal nerve fiber layer thickness (cpRNFL), standard deviation (SD), Not Applicable (NA).

**Table 9:** Comparison of optic disc parameters in children across studies.

	Age	Participants	Race	Equipment	Disc area	Cup area	CDR
	Range	n			Mean±SD (mm^2^) (5th–95th percentile)		ratio
Current Study	8	349	Japanese	RS-3000 Advance2	2.07 ± 0.37 (1.52–2.71)	0.56 ± 0.31 (0.15–1.11)	0.26 ± 0.12 (0.09–0.47)
Bueno-Gimeno et al [18]	6–17	293	Caucasian	Cirrus-HD	1.88 ± 0.34	NA	0.34 ± 0.19
Dave et al [19]	5–18	126	Indian	Spectralis	1.7 ± 0.6	NA	NA
El-Dairi et al [6]	3–17	286	Caucasian, African American	Stratus	2.42 (1.74–3.21)	0.47 (0–1.14)	0.20 (0–0.46)
Yabas Kiziloglu et al [20]	5–17	128	Turkish	iVue 100	2.30 ± 0.42	0.09 ± 0.10	0.20 ± 0.13
Huynh et al [21]	11–14	2054	Caucasian, East Asian Middle Eastern, Others	Stratus	2.34 ± 0.41	0.46 ± 0.32	0.21 ± 0.14

Abbreviations: cup-to-disc ratio (CDR), standard deviation (SD), Not Applicable (NA).

The neuroretinal rim of the normal eye is thickest in the inferior quadrant, followed by the superior, nasal, and temporal quadrants, as reported by the inferior>superior>nasal>temporal (ISNT) rule [22]. In this study, however, the thickness decreased from the superior quadrant to the inferior, temporal and finally the nasal quadrants. Previous reports on children have shown similar results in that the thickness in either the inferior or superior quadrant was greatest, whereas that in either the nasal or temporal quadrant was lowest [9,17–19]. Dave et al. [19] reported that only 30 of 126 eyes (23.8%) in children followed the ISNT rule, whereas 52% of eyes followed the inferior>superior>temporal (IST) rule.

The disc area and cup area differ from report to report, but this may be due to the OCT devices used, the ethnicities of the participants, and the sizes of the included sample [23]. The results may also depending on whether or not corrections are made for the ocular magnification effect [15].

In this study, the temporal, 8 o’clock cpRNFL thickness was significantly positively correlated with the AL, whereas the inferior, 6 o’clock cpRNFL thickness was significantly negatively correlated with the AL. In a study involving 6–17-year-old children, Bueno-Gimeno et al. [18] compared the characteristics among long, medium, and short AL groups and reported that the long AL group had a significantly thinner RNFL than did the medium and short AL groups in all except the temporal side. In a study of children aged 4–18 years, Söhnel et al. [8] reported a significant negative correlation between AL and the RNFL thickness in all except the temporal side. In a study involving children aged 6–8 years, Zhang et al. [7] reported similar results to those obtained in this study, demonstrating a positive correlation between AL and the RNFL thickness in the temporal side and a negative correlation between AL and the RNFL thickness in the nasal, superior, and inferior sides.

The temporal quadrant is where the papillomacular bundle is located, and optic nerve fibers are located mostly near the horizontal meridian from the macula to the optic disc. In adults, the RNFL is reportedly thicker in the temporal quadrant because the convergence of the papillomacular bundle toward the temporal horizon due to axial elongation causes a rearrangement of the RNFL [24,25]. In a study of adults with myopia, Choi et al. reported that the RNFL thickened in the superior quadrants and became thinner in the inferior quadrant due to a decrease in the fovea-disc angle [26]. In another study involving adults, Yamashita et al. reported a negative correlation between AL and the temporal major arterial angle and between AL and the peak angle of the nerve fiber layer thickness; however, both of these angles increased as the temporal cpRNFL thickness and the AL increased [27]. In this study, the temporal cpRNFL thickness increased as the AL increased, but further investigations of the relationships between the thickness of the cpRNFL and the foveal location and artery and peak RNFL angle in children are needed.

In this study, the cpRNFL thickened with myopia at 8 o’clock and thinned at 6 o’clock. In a study on 6–17-year-olds, Al-Hadadd et al. reported that the RNFL was significantly thinner on all but the temporal side as the refractive error indicated a greater degree of myopia [17]. Bueno-Gimeno et al. reported that the RNFL was significantly thinner in children with high myopia than in those with moderate or mild myopia in all except the temporal side [18]. In contrast, Söhnel et al. reported no correlation between refractive error and the RNFL thickness in children [8].

In this study, multivariable regression analysis showed a significant positive correlation of the disc area with AL and the whole cpRNFL thickness. In a pediatric study, Bueno-Gimeno et al. reported that AL, SE, and age were not associated with disc area or cup area, which differs partially from our results [18]. Few studies have examined the relationship between the cpRNFL thickness and disc area in children, while Yamashita et al. reported a positive correlation between the disc area and whole RNFL thickness in adults [25]. Jonas et al. reported that large optic disc diameters may indicate the presence of many retinal nerve fibers [22]. In addition, when performing measurements, the OCT device uses a scan circle with a diameter of 3.24 mm around the optic disc, which may reflect a greater cpRNFL thickness close to the optic disc, as the margin between the disc edge and the scan circle decreases as the optic disc diameter increases [28].

In this study, multivariable regression analysis showed a significant positive correlation between sex and cpRNFL thickness in the 10 o’clock and 11 o’clock sectors and a significant negative correlation between sex and the cpRNFL thickness in the 12 o’clock sector, indicating that girls had a thicker cpRNFL on the superotemporal side, whereas boys had thicker cpRNFL on the superior side. Furthermore, the disc area was significantly larger in boys. Wagner et al. reported that in adults, the peak angle of the RNFL thickness in the temporal side was smaller in women than in men [29]. Alasil et al. reported no sex difference in RNFL thickness in their study of 9–86-year-olds [30], whereas Huynh et al. reported sex differences in the thickness of the RNFL, with significantly thicker RNFLs at the 9 o’clock and 12 o’clock positions for boys and at the 4 o’clock, 7 o’clock and 10 o’clock positions for girls [21]. They also reported that disc diameter, disc area, and rim area were greater in girls than in boys, whereas the cup-to-disc ratio was greater in boys. In that report, age, AL, and refractive error were greater than those in our study, and several ethnic groups were included, which may explain why the results differed from those in this study.

In this study, we investigated the associations of the cpRNFL thickness and disc parameters with AL, SE, and sex in 8-year-old Japanese children. A limitation of this study is that cycloplegic drops could not be used; therefore, the association with refractive error may differ from that in other reports. A study of children aged 6–8 years reported that the use of cycloplegic drops caused refractive error changes of 0.50D or greater in 60.4% of hyperopic eyes and 37.5% of myopic eyes [31]. The correlation between the cpRNFL and refractive error may have been underestimated by not using cycloplegia drops in this study. In this study, the data from the left eye was used for analysis when the right eye was not available. However, the cpRNFL thickness differed between the two eyes in the 1 o’clock, 2 o’clock, 8 o’clock, 10 o’clock, and 11 o’clock sectors (S2 Table). Multivariable regression analysis was performed on the data from the right and left eyes of the included participants in separate groups, but the trends were similar to those observed for the entire participant group (S3–6 Tables). Considering the possibility that the cpRNFL thickness is asymmetric between the left and right eyes, it was considered better to perform the analysis without including both the left and right eyes. Many participants were excluded from this study because it was difficult for these young patients to cooperate with the measurement procedures. In addition, the excluded group had a significantly greater degree of myopia and significantly lower uncorrected visual acuity (S7 Table). The narrower range of refractive error in the included group may have resulted in an underestimation of the effect of the refractive error on cpRNFL thickness. However, most of the reasons for exclusion were related to the OCT images, such as gaze maintenance, eyelid opening, and posture during the examination. The tight examination schedule may not have allowed participants sufficient time to cooperate with the ophthalmologic examination. Thus, sample bias was not considered a significant effect.

Regarding the effect of age, a study on Taiwanese adults reported that the RNFL thickness decreased by 4.97 µm per year [32]. While some reports have indicated that the thickness of the RNFL in childhood does not significantly change with age [8,21,33], others report an increase with age [7]. Other biological data and lifestyle habits could also have affected the results, as it has been reported that children with higher body mass index (BMI) have a thicker RNFL [7] and that those who engage with screens for longer durations have a thinner nasal RNFL [8]. The JECS is scheduled to continue until 2027, and a longitudinal survey is underway to determine the changes in the RNFL over time. The JECS also includes physical measurements, blood tests, physical and dental examinations, and questionnaires on parents’ health status and lifestyles. In the future, further analyses of the factors influencing the cpRNFL thickness in children will be conducted by examining its relationship with these data.

## Conclusion

In the 8-year-old Japanese children in this study, the cpRNFL thickness was thinner at 6 o’clock and thicker at 8 o’clock due to AL elongation and myopia. Boys had a thicker superior cpRNFLs, whereas girls had a thicker superotemporal cpRNFLs. A significant positive correlation was observed between disc area and cpRNFL thickness. We plan to examine the relationships between the cpRNFL thickness and physical and blood test data as well as the changes over time in factors associated with the thickness of the cpRNFL.

## Supporting information

S1 Table The circumpapillary retinal nerve fiber layer thickness comparison between quadrants. (DOCX)

S2 Table Comparison of left and right eye differences in cpRNFL thickness in the included groups. (DOCX)

S3 Table Multivariable regression analysis of cpRNFL thickness (right eye of included group). (DOCX)

S4 Table Multivariable regression analysis of optic disc parameters (right eye of included group). (DOCX)

S5 Table Multivariable regression analysis of cpRNFL thickness (left eye of included group). (DOCX)

S6 Table Multivariable regression analysis of optic disc parameters (left eye of included group). (DOCX)

S7 Table Comparison of included and excluded groups. (DOCX)
