# Supplementary material for: Factors associated with optic disc parameters and circumpapillary retinal nerve fiber layer thickness in 8-year-old children: The Yamanashi Adjunct Study of the Japan Environment and Children’s Study
Source: PLoS One. 2025 Aug 20;20(8):e0330335. doi: 10.1371/journal.pone.0330335 (PMC12367147; doi:10.1371/journal.pone.0330335)
Supplement: S1 Table — (DOCX) [file pone.0330335.s001.docx]

**S1 Table: The circumpapillary retinal nerve fiber layer thickness comparison between quadrants.**

|  | Inferior | Nasal | Superior |
| --- | --- | --- | --- |
| Nasal | < 2e-16 | - | - |
| Superior | 1.00 | < 2e-16 | - |
| Temporal | < 2e-16 | < 2e-16 | < 2e-16 |

Data are P-values from one-way analysis of variance.
