# Supplementary material for: Factors associated with optic disc parameters and circumpapillary retinal nerve fiber layer thickness in 8-year-old children: The Yamanashi Adjunct Study of the Japan Environment and Children’s Study
Source: PLoS One. 2025 Aug 20;20(8):e0330335. doi: 10.1371/journal.pone.0330335 (PMC12367147; doi:10.1371/journal.pone.0330335)
Supplement: S2 Table — (DOCX) [file pone.0330335.s002.docx]

**S2 Table: Comparison of left and right eye differences in cpRNFL thickness in the included groups.**

|  | **Right eye**  **(n=213)** | **Left eye**  **(n=136)** | **P ^a^** |
| --- | --- | --- | --- |
| cpRNFL Thickness (µm)  Whole | 96.5±9.0 (82–113) | 95.5±8.8 (79–109) | 0.41 |
| 1 o’clock | 104.3±20.5 (70–136) | 115.1±26.3 (68–153) | <0.001 |
| 2 o’clock | 78.7±19.4 (50–118) | 70.4±16.5 (45–98) | <0.001 |
| 3 o’clock | 50.9±9.2 (38–69) | 49.2±8.0 (37–62) | 0.15 |
| 4 o’clock | 62.0±14.0 (41–88) | 61.1±15.1 (42–86) | 0.45 |
| 5 o’clock | 93.8±19.8 (63–127) | 94.7±19.4 (66–132) | 0.78 |
| 6 o’clock | 130.2±23.6 (91–167) | 131.2±23.2 (98–169) | 0.75 |
| 7 o’clock | 146.4±22.9 (107–182) | 144.3±21.9 (107–179) | 0.32 |
| 8 o’clock | 76.9±14.2 (57–104) | 72.9±14.0 (55–103) | 0.002 |
| 9 o’clock | 60.2±9.5 (48–74) | 60.0±10.2 (47–78) | 0.67 |
| 10 o’clock | 88.3±13.2 (67–110) | 85.0±13.0 (65–105) | 0.03 |
| 11 o’clock | 144.8±22.9 (102–179) | 137.1±23.6 (95–173) | 0.003 |
| 12 o’clock | 121.0±28.0 (79–171) | 124.9±27.4 (86–174) | 0.26 |

The included groups (n=349) were analyzed by right eye (n=213) and left eye (n=136).

Measurements are presented as mean ± standard deviation (5–95th percentile).

Abbreviations: circumpapillary retinal nerve fiber layer thickness(cpRNFL).

^a^ Mann-Whitney U test
