# Supplementary material for: Factors associated with optic disc parameters and circumpapillary retinal nerve fiber layer thickness in 8-year-old children: The Yamanashi Adjunct Study of the Japan Environment and Children’s Study
Source: PLoS One. 2025 Aug 20;20(8):e0330335. doi: 10.1371/journal.pone.0330335 (PMC12367147; doi:10.1371/journal.pone.0330335)
Supplement: S3 Table — (DOCX) [file pone.0330335.s003.docx]

**S3 Table: Multivariable regression analysis of cpRNFL thickness (right eye of included group).**

|  | **AL** | | | | | **Sex** | | | | | **DIsc area** | | | | |
| --- | --- | --- | --- | --- | --- | --- | --- | --- | --- | --- | --- | --- | --- | --- | --- |
| **cpRNFL**  **Thickness** | **B ^a^** | **95%CI of B** | | **β ^b^** | **P** | **B ^a^** | **95%CI of B** | | **β ^b^** | **P** | **B ^a^** | **95%CI of B** | | **β ^b^** | **P** |
| 1 o’clock | 3.95 | 0.17 | 7.33 | 0.15 | 0.12 | -2.67 | -8.50 | 3.16 | -0.07 | 1.00 | 6.91 | -0.81 | 14.63 | 0.12 | 0.21 |
| 2 o’clock | -1.29 | -4.97 | 2.38 | -0.05 | 1.00 | -2.30 | -7.97 | 3.37 | -0.06 | 1.00 | 3.68 | -3.83 | 11.18 | 0.07 | 0.99 |
| 3 o’clock | 0.44 | -1.29 | 2.17 | 0.04 | 1.00 | -1.08 | -3.75 | 1.58 | -0.06 | 1.00 | -4.10 | -7.62 | -0.57 | -0.17 | 0.06 |
| 4 o’clock | 1.46 | -1.21 | 4.12 | 0.08 | 0.84 | -0.005 | -4.11 | 4.10 | -0.0002 | 1.00 | 0.20 | -5.24 | 5.64 | 0.005 | 1.00 |
| 5 o’clock | -1.26 | -5.04 | 2.51 | -0.05 | 1.00 | 0.20 | -5.61 | 6.01 | 0.005 | 1.00 | 2.46 | -5.24 | 10.15 | 0.05 | 1.00 |
| 6 o’clock | -6.78 | -11.17 | -2.38 | -0.23 | 0.006 | -4.35 | -11.13 | 2.42 | -0.09 | 0.60 | 7.04 | -1.93 | 16.01 | 0.11 | 0.36 |
| 7 o’clock | 0.65 | -3.69 | 4.98 | 0.02 | 1.00 | 3.74 | -2.93 | 10.42 | 0.08 | 0.81 | 4.75 | -4.09 | 13.59 | 0.08 | 0.87 |
| 8 o’clock | 5.73 | 3.19 | 8.27 | 0.32 | <0.001 | 0.77 | -3.15 | 4.68 | 0.03 | 1.00 | 3.59 | -1.59 | 8.77 | 0.09 | 0.51 |
| 9 o’clock | 2.27 | 0.48 | 4.06 | 0.19 | 0.03 | 0.01 | -2.75 | 2.77 | 0.0006 | 1.00 | -1.39 | -5.04 | 2.26 | -0.05 | 1.00 |
| 10 o’clock | 2.12 | -0.35 | 4,60 | 0.13 | 0.27 | 4.99 | 1,18 | 8.80 | 0.19 | 0.03 | 2,46 | -2.59 | 7.50 | 0.07 | 0.99 |
| 11 o’clock | 1.63 | -2.58 | 5.85 | 0.06 | 1.00 | 7.84 | 1.35 | 14.33 | 0.17 | 0.03 | 13.20 | 4.60 | 21.79 | 0.21 | 0.006 |
| 12 o’clock | 0.54 | -4.61 | 5.68 | 0.02 | 1.00 | -13.82 | -21.75 | -5,89 | -0.25 | <0.001 | 3.91 | -6.59 | 14.41 | 0.05 | 1.00 |

Sex was analyzed by assigning boys a value of 0 and girls a value of 1.

Abbreviations: circumpapillary retinal nerve fiber layer thickness(cpRNFL), axial length(AL), confidence interval(CI).

^a^ Nonstandardized Regression Coefficient B.

^b^ Standardized Regression Coefficient β.
