# Supplementary material for: Factors associated with optic disc parameters and circumpapillary retinal nerve fiber layer thickness in 8-year-old children: The Yamanashi Adjunct Study of the Japan Environment and Children’s Study
Source: PLoS One. 2025 Aug 20;20(8):e0330335. doi: 10.1371/journal.pone.0330335 (PMC12367147; doi:10.1371/journal.pone.0330335)
Supplement: S5 Table — (DOCX) [file pone.0330335.s005.docx]

**S5 Table: Multivariable regression analysis of cpRNFL thickness (left eye of included group).**

Sex was analyzed by assigning boys a value of 0 and girls a value of 1.

Abbreviations: circumpapillary retinal nerve fiber layer thickness(cpRNFL), axial length(AL), confidence interval(CI).

^a^ Nonstandardized Regression Coefficient B.

^b^ Standardized Regression Coefficient β.

|  | **AL** | | | | | **Sex** | | | | | **DIsc area** | | | | |
| --- | --- | --- | --- | --- | --- | --- | --- | --- | --- | --- | --- | --- | --- | --- | --- |
| **cpRNFL**  **Thickness** | **B ^a^** | **95%CI of B** | | **β ^b^** | **P** | **B ^a^** | **95%CI of B** | | **β ^b^** | **P** | **B ^a^** | **95%CI of B** | | **β ^b^** | **P** |
| 1 o’clock | -3.72 | -10.38 | 2.94 | -0.10 | 0.81 | -8.19 | -17.52 | 1.14 | -0.16 | 0.24 | 10.21 | -2.77 | 23.18 | 0.14 | 0.36 |
| 2 o’clock | -1.82 | -6.05 | 2.42 | -0.08 | 1.00 | -2.76 | -8.69 | 3.17 | -0.08 | 1.00 | 4.79 | -3.46 | 13.04 | 0.10 | 0.99 |
| 3 o’clock | -0.10 | -3.05 | 1.07 | -0.09 | 1.00 | 0.69 | -2.20 | 3,58 | 0.04 | 1.00 | 0.78 | -3.24 | 4,80 | 0.03 | 0.06 |
| 4 o’clock | -1.89 | -5.76 | 1.98 | -0.09 | 0.48 | -0.66 | -6.08 | 4.77 | -0.02 | 1.00 | 4.51 | -3.03 | 12.05 | 0.11 | 1.00 |
| 5 o’clock | -4.13 | -9.09 | 0.84 | -0.15 | 1.00 | -3.95 | -10.91 | 3.01 | -0.10 | 1.00 | 1.92 | -7.76 | 11.60 | 0.03 | 1.00 |
| 6 o’clock | -5.68 | -11.55 | 0.19 | -0.18 | 0.009 | -3.62 | -11.85 | 4.61 | -0.08 | 0.54 | 10.02 | -1.42 | 21.47 | 0.15 | 0.36 |
| 7 o’clock | -3.26 | -8.77 | 2.26 | -0.11 | 0.69 | 4.39 | -3.34 | 12.12 | 0.10 | 0.69 | 12.36 | 1.61 | 23.10 | 0.20 | 0.87 |
| 8 o’clock | 2.76 | -0.81 | 6.33 | 0.14 | 0.36 | 0.36 | -4.65 | 5,36 | 0.01 | 1.00 | 2,77 | -4.19 | 9.73 | 0.07 | 1.00 |
| 9 o’clock | -0.85 | -3.44 | 1.74 | -0.06 | 1.00 | -0.86 | -4.50 | 2.77 | -0.04 | 1.00 | 5.26 | 0.20 | 10.31 | 0.18 | 0.12 |
| 10 o’clock | 1.90 | -1.34 | 5.14 | 0.10 | 0.72 | 5.60 | 1.07 | 10.14 | 0.21 | 0.03 | 7.26 | 0.96 | 13.57 | 0.20 | 0.06 |
| 11 o’clock | -4.89 | -10.69 | 0.91 | -0.15 | 0.27 | 4.49 | -3.64 | 12.61 | 0.09 | 0.81 | 19.92 | 8.62 | 31.22 | 0.30 | 0.001 |
| 12 o’clock | -1.58 | -8.38 | 5.23 | -0.04 | 1.00 | -14.45 | -23.99 | -4.91 | -0.26 | 0.009 | 8.09 | -5.17 | 21.35 | 0.10 | 0.66 |
