# Supplementary material for: Factors associated with optic disc parameters and circumpapillary retinal nerve fiber layer thickness in 8-year-old children: The Yamanashi Adjunct Study of the Japan Environment and Children’s Study
Source: PLoS One. 2025 Aug 20;20(8):e0330335. doi: 10.1371/journal.pone.0330335 (PMC12367147; doi:10.1371/journal.pone.0330335)
Supplement: S6 Table — (DOCX) [file pone.0330335.s006.docx]

**S6 Table: Multivariable regression analysis of optic disc parameters (left eye of included group).**

|  | **AL** | | | | | **Sex** | | | | | **Whole cpRNFL thickness** | | | | |
| --- | --- | --- | --- | --- | --- | --- | --- | --- | --- | --- | --- | --- | --- | --- | --- |
|  | **B ^a^** | **95%CI of B** | | **β ^b^** | **P** | **B ^a^** | **95%CI of B** | | **β ^b^** | **P** | **B ^a^** | **95%CI of B** | | **β ^b^** | **P** |
| Disc area | 0.12 | 0.04 | 0.20 | 0.25 | 0.01 | -0.001 | -0.12 | 0.12 | -0.002 | 1.00 | 0.01 | 0.005 | 0.02 | 0.28 | 0.002 |
| Cup area | 0.06 | -0.02 | 0.13 | 0.14 | 0.39 | -0.008 | -0.11 | 0.10 | -0.01 | 1.00 | 0.004 | -0.002 | 0.009 | 0.11 | 0.60 |
| CDR | 0.01 | -0.02 | 0.04 | 0.08 | 1.00 | 0.005 | -0.04 | 0.05 | 0.02 | 1.00 | 0.0005 | -0.002 | 0.003 | 0.04 | 1.00 |

Sex was analyzed by assigning boys a value of 0 and girls a value of 1.

Abbreviations: axial length(AL), circumpapillary retinal nerve fiber layer thickness(cpRNFL), confidence interval(CI), cup-to-disc ratio (CDR).

^a^ Nonstandardized Regression Coefficient B.

^b^ Standardized Regression Coefficient β.
