# Supplementary material for: Factors associated with optic disc parameters and circumpapillary retinal nerve fiber layer thickness in 8-year-old children: The Yamanashi Adjunct Study of the Japan Environment and Children’s Study
Source: PLoS One. 2025 Aug 20;20(8):e0330335. doi: 10.1371/journal.pone.0330335 (PMC12367147; doi:10.1371/journal.pone.0330335)
Supplement: S7 Table — (DOCX) [file pone.0330335.s007.docx]

**S7 Table: Comparison of included and excluded groups.**

|  | Included  (n=349) | Excluded ^a^  (n=178) | Excluded ^b^  (n=32) | P ^c^ |
| --- | --- | --- | --- | --- |
| AL (mm) | 23.11±0.77  (21.90–24.37) | 23.20±0.82  (21.91–24.58) | 23.00±0.97  (21.64–24.48) | 0.31 |
| SE (D) | -0.35±0.94  (-1.88–0.63) | -0.70±1.14  (-3.00–+0.63) | -2.07±3.41  (-9.50–+2.29) | <0.001 |
| Uncorrected logMAR | 0.06±0.18  (0.00–0.52) | 0.13±0.26  (0.00–0.72) | 0.33±0.36  (0.00–1.00) | <0.001 |
|  | **n** | **n** | **n** | **P ^d^** |
| Boy  Girl | 182  167 | 83  95 | 11  21 | 0.10 |

Measurements are presented as mean ± standard deviation (5–95th percentile).

Abbreviations: axial length (AL), spherical equivalent (SE).

^a^ excluded participants; 10 participants who did not cooperate with optical coherence tomography measurements or AL imaging and 168 participants who had poor image quality due to blinking, poor fixation or poor postural retention.

^b^ excluded participants; 19 participants with visual acuity worse than logMAR 0 and 13 participants with strabismus, amblyopia, or aniseikonia.

^c^ one-way analysis of variance

^d^ χ^2^ test.
